# Supplementary material for: C16-ceramide is a natural regulatory ligand of p53 in cellular stress response
Source: Nat Commun. 2018 Oct 8;9:4149. doi: 10.1038/s41467-018-06650-y (PMC6175828; doi:10.1038/s41467-018-06650-y)

Supplementary information for

C_{16}-ceramide is a natural regulatory ligand of p53 in cellular stress response

Fekry et al.

This PDF file includes:

Supplementary Figures 1 to 12

Supplementary Table 1

Supplementary References

Full Blot Scans

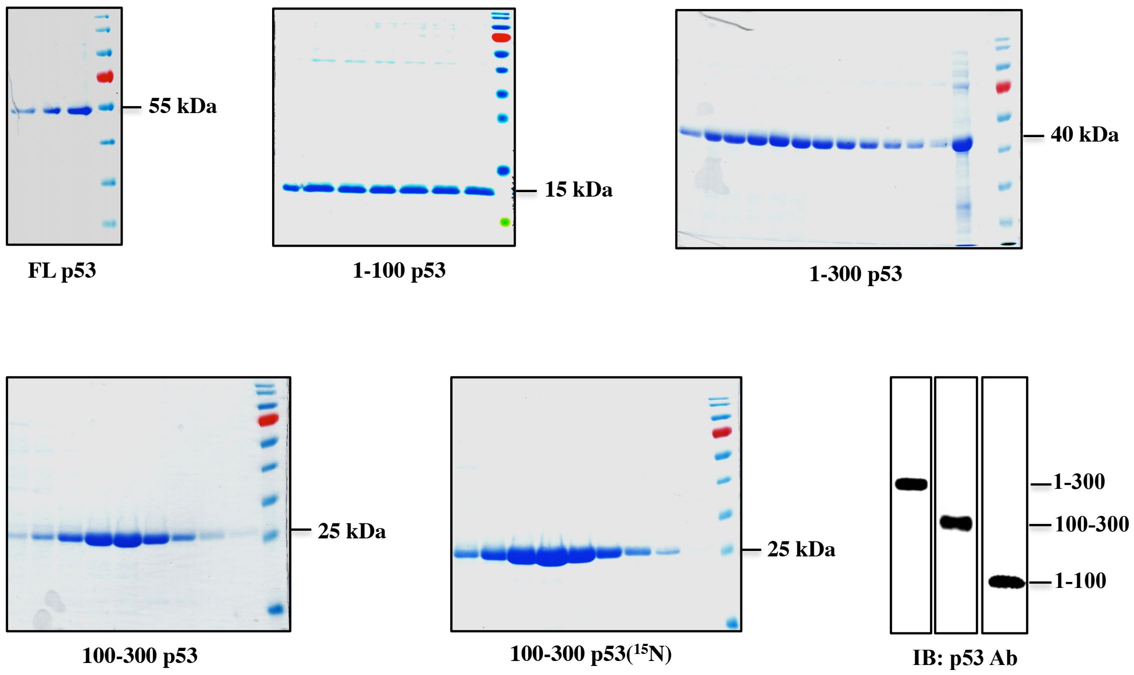

Supplementary Figure 1. Purification and characterization of the full-length p53 (FLp53) and its truncated variants used in pull-down, membrane-binding and ubiquitination assays, and pacFA ceramide modification.

Coomassie stained gels (SDS-PAGE) show purified proteins; Western blots show pooled fractions. Numbers indicate the first and the last amino acid residues (according to the full-length protein sequence) present in each construct.

D-e-C18-Ceramide-14-Pyridinium Bromide (PC_{18})

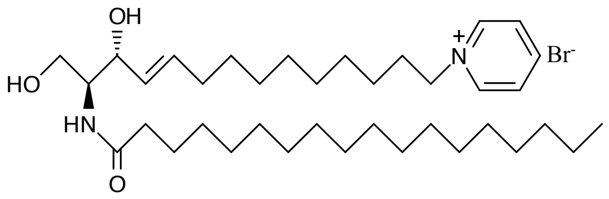

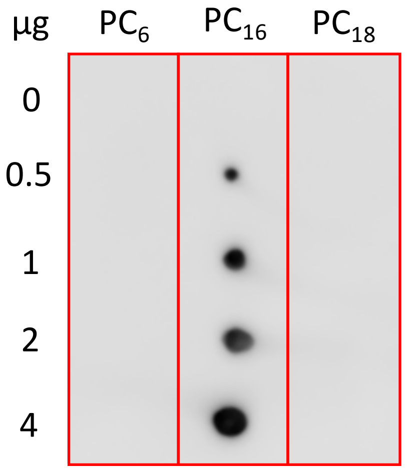

Supplementary Figure 2. p53 does not bind C_{18}-ceramide with the pyridinium ring on sphingoid base. Left panel, structure of a soluble ceramide compound with the C_{18}-acyl chain and the pyridinium ring on the sphingoid base. Right panel, binding of purified p53 to a panel of pyridinium ceramides with different acyl chains (C_6, C_{16} and C_{18}) immobilized on a PVDF membrane. Amounts of compounds in respective spots are indicated; bound p53 was detected with specific antibody.

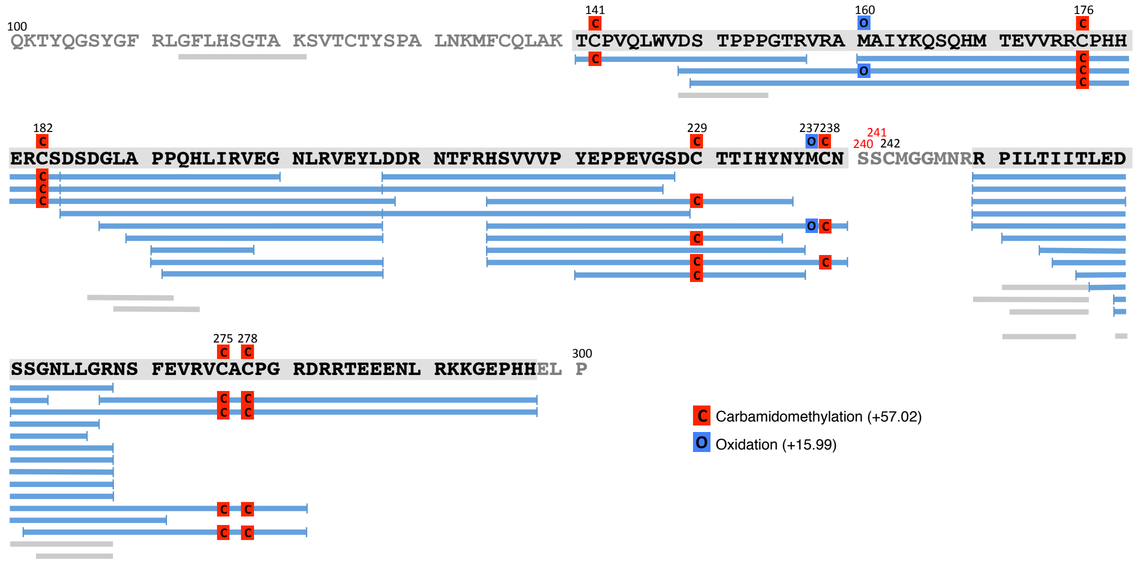

Supplementary Figure 3. Peptides and post-translational modifications identified by PEAKS software in the p53 DNA-binding domain UV-irradiated in the absence of pacFA-ceramide. PEAKS analysis identified only carbamidomethylated and oxidized peptides in the p53 DBD sample processed without photoactivatable ceramide and no peptides with 518.46 Da modification.

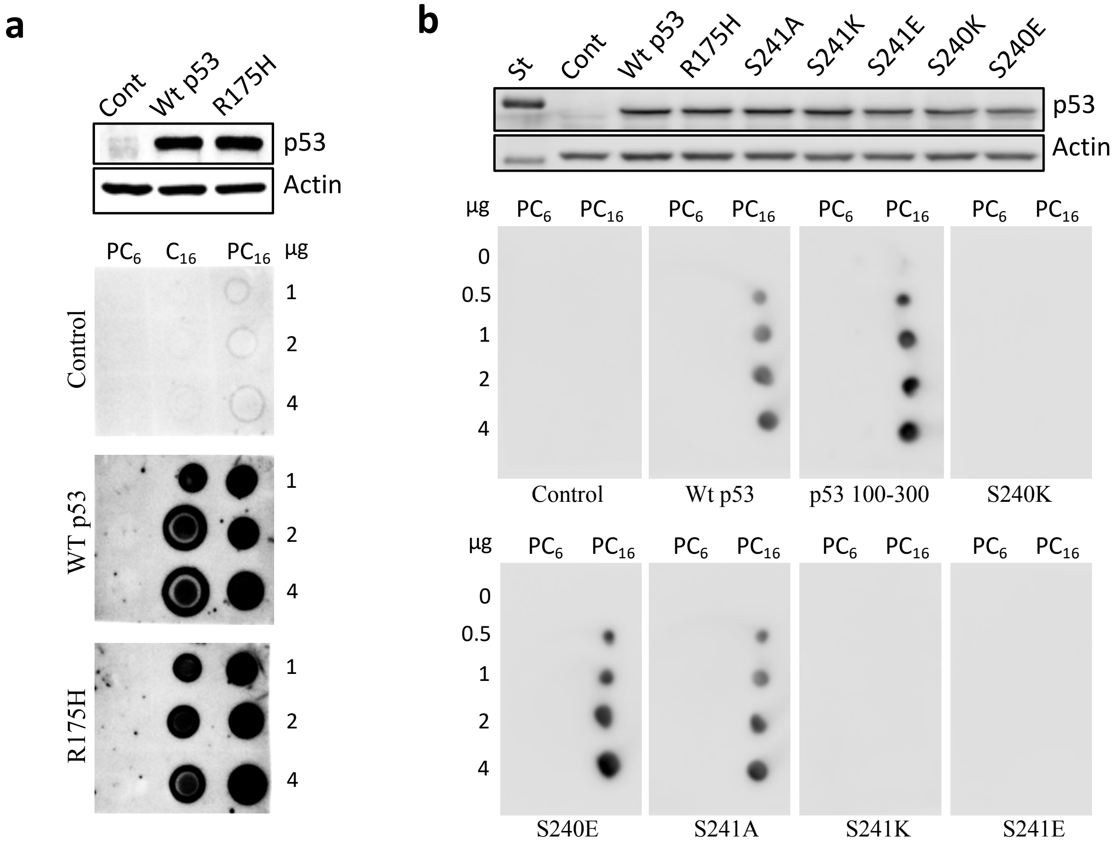

Supplementary Figure 4. Point mutation effects on the p53-ceramide binding. (**a**) The DNA-binding deficient R175H p53 mutant binds both natural (C_{16}) and pyridinium (PC_{16}) ceramides as efficiently as the WT p53. A representative membrane binding assay is shown. (**b**) The p53 point mutants S240K, S241K and S241E are unable to bind PC_{16}, while S240E and S241A mutants are efficient binders of PC_{16}. Representative binding assays are shown; PC_6 is used as a negative control.

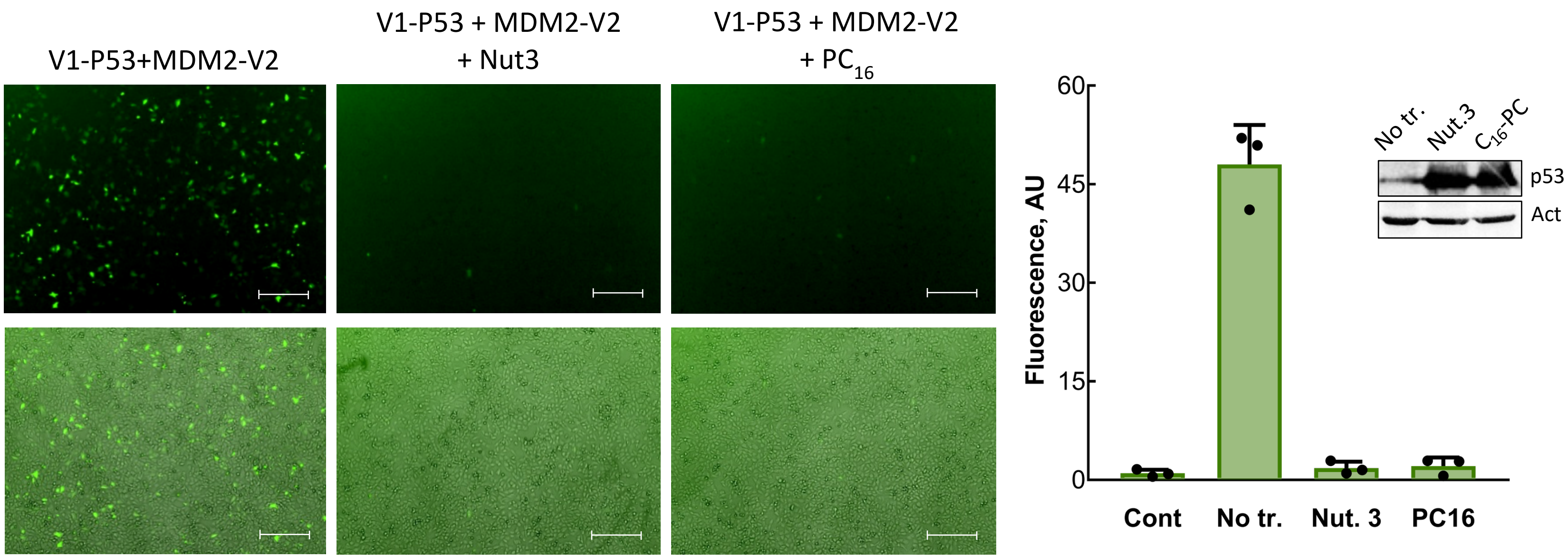

Supplementary Figure 5. Detection and quantification of p53-Mdm2 complementation reactions by fluorescence microscopy. Low magnification (x4) fluorescence images (top panel) and overlay with the corresponding phase-contrast images (bottom panel) show fluorescence complementation in PC-3 cells co-transfected with V_1-p53 and MDM2-V_2. Scale bar represents 200 µm. Cell treatment with 50 µM Nutlin-3, or 5 µM PC_{16} prevents fluorescence complementation without affecting their density/viability. Graph on the right represents quantification of the fluorescence in control (non-transfected), as well as V_1-p53 and MDM2-V_2-transfected cells, both untreated and treated with Nutlin-3 and PC_{16}. Fluorescence analysis was accomplished using Cytell Cell Imaging System (GE Healthcare), where fluorescence for each replicate was calculated as an average of measurements in 20 randomly selected fields of the same well. Experiments were performed in triplicate. Inset shows p53 levels in the non-treated and treated cells.

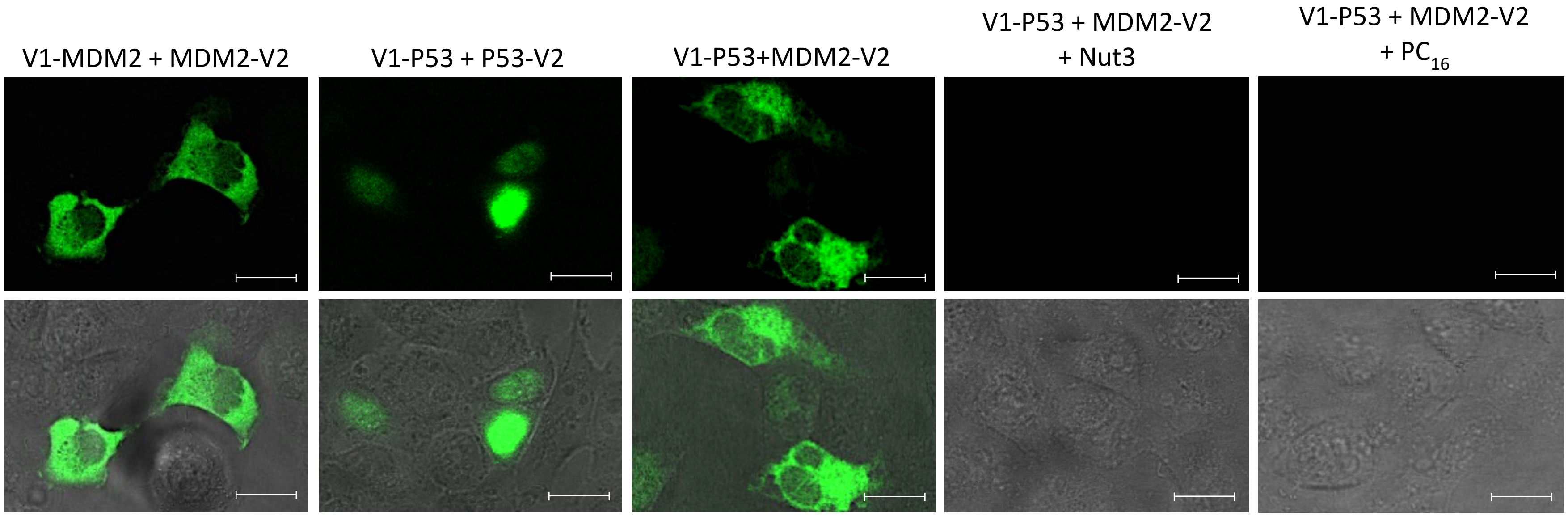

Supplementary Figure 6. Detection and subcellular localization of Mdm2-MDM2, p53-p53, and p53-Mdm2 complementation reactions by fluorescence microscopy.

High magnification (x40) fluorescence images (top panel) and overlay with the corresponding phase-contrast images (bottom panel) show fluorescence complementation in PC-3 cells co-transfected with V_1-MDM2 + MDM2-V_2, V_1-p53 + p53-V_2 and V_1-p53 + MDM2-V_2. Treatment of V_1-p53 + MDM2-V_2 transfected cells with 50 µM Nutlin-3, or 5 µM PC_{16} efficiently disrupt V_1-p53 + MDM2-V_2 fluorescence complementation without affecting cell density or morphology. Scale bar represents 20 µm.

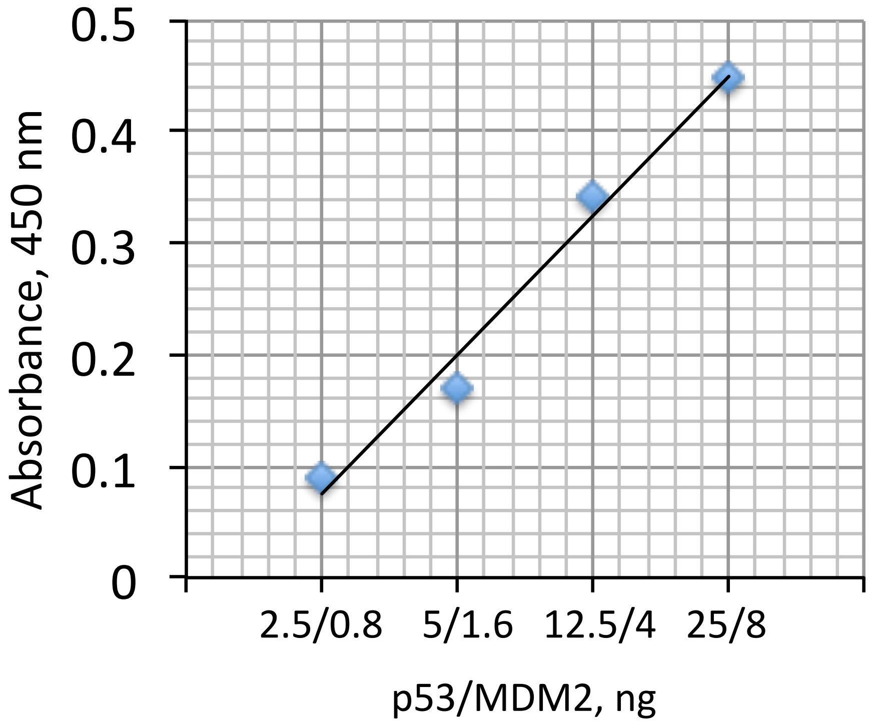

Supplementary Figure 7. Standard curve for the measurement of p53-MDM2 complex by ELISA. Polyclonal p53 antibody was used as a capture antibody. The standard curve was obtained by adding indicated amounts of pre-mixed purified recombinant p53 and MDM2 to the plate. MDM2 monoclonal antibody and secondary HRP conjugated antibody were used for detection. The plate was developed with TMB and absorbance registered at 450 nm.

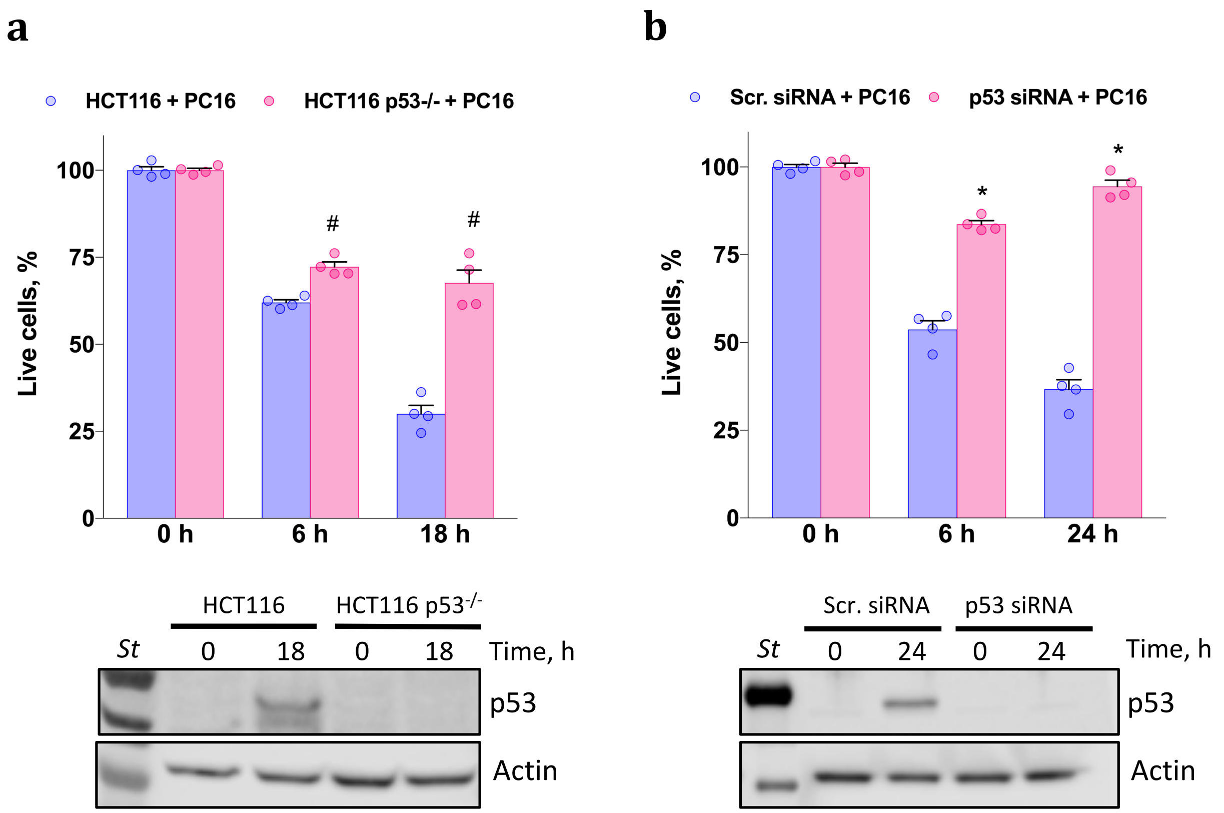

Supplementary Figure 8. Absence of p53 partially protects cancer cells from PC_{16} toxicity. (**a**) HCT116 cells are more sensitive to the cytotoxic effects of PC_{16} than isogenic HCT116 $p53^{-/-}$ cells. Error bars represent ±SE, n=4 (#, P<0.0001). Lower panel shows p53 levels at indicated time points. (**b**) The siRNA silencing of p53 results in a significant rescue of HepG2 sells from PC_{16} cytotoxicity. Error bars represent ±SE, n=4 (*, P<0.001). Lower panel shows p53 levels at indicated time points. Student's t-test was used for statistical analysis in both cell lines.

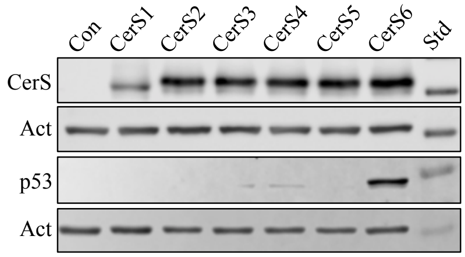

Enzyme	Specificity	Enzyme	Specificity
CerS1	C_{18}	*CerS4*	C_{18}-C_{20}
CerS2	C_{20}-C_{26}	*CerS5*	C_{14}-C_{16}
CerS3	C_{24}-C_{36}	*CerS6*	C_{14}-C_{16}

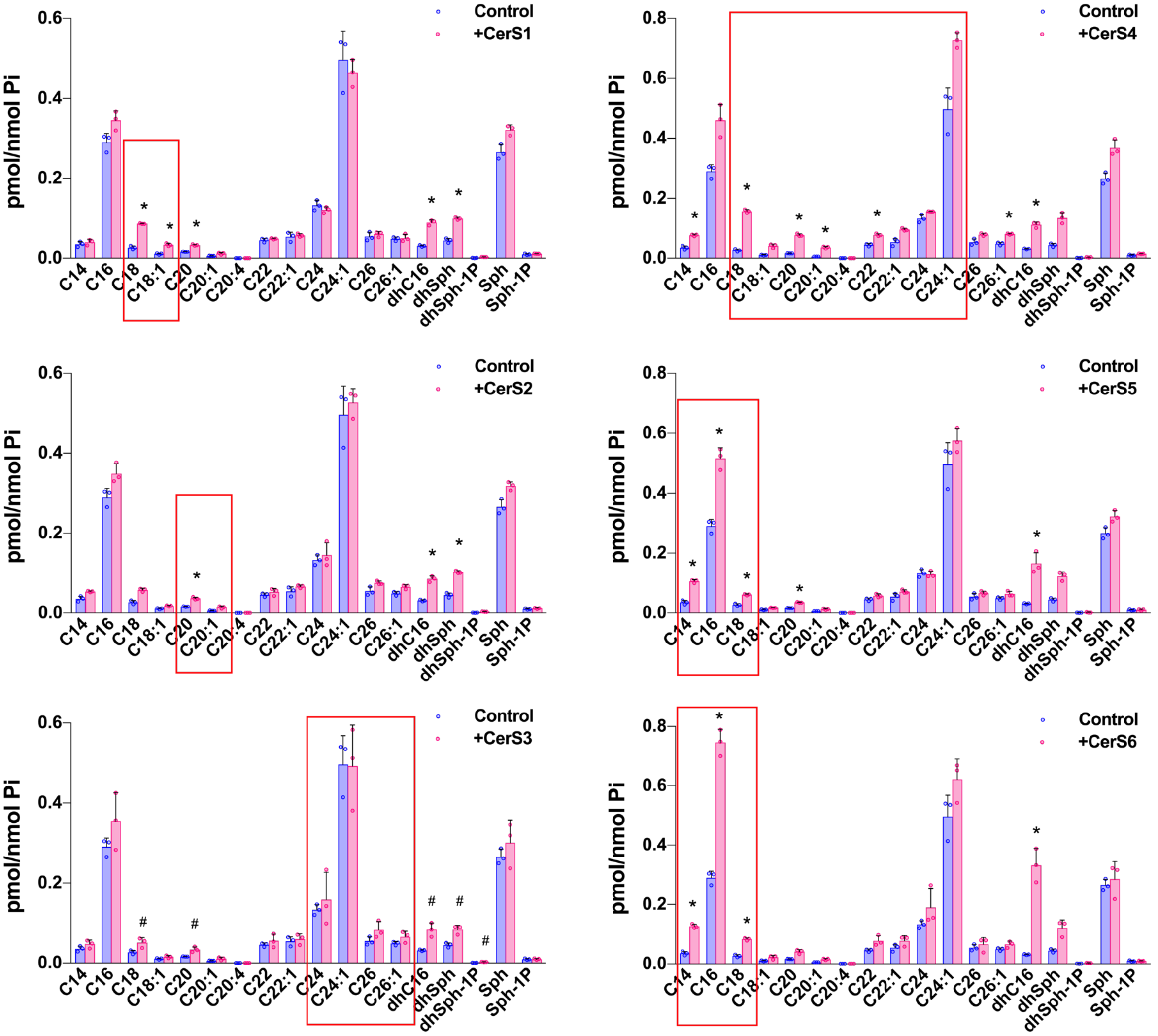

Supplementary Figure 9. Transient transfection of A549 cells with individual ceramide synthases, CerS1-5, did not induce accumulation of the p53 protein, while transfection with CerS6 results in a significant elevation of p53. Western blot shows comparable expression levels of the individual HA-tagged CerS2-6 and Flag-tagged CerS1 upon transient transfection. Significant accumulation of p53 was found only in CerS6 transfected cells. Table on the top shows

preference of ceramide synthases towards specific acyl chain lengths (summary from published data[1-5]). Bar graphs represent ceramide species measurements in A549 cells transfected with individual ceramide synthases 36 h post-transfection.

Averages of three individual transfections ±SD for each CerS are shown. * denotes statistically significant differences with $P<0.001$, # denotes statistically significant differences with $P<0.05$. Student t-test was used for statistical analysis. Red rectangles mark specific ceramide species produced by the corresponding CerS.

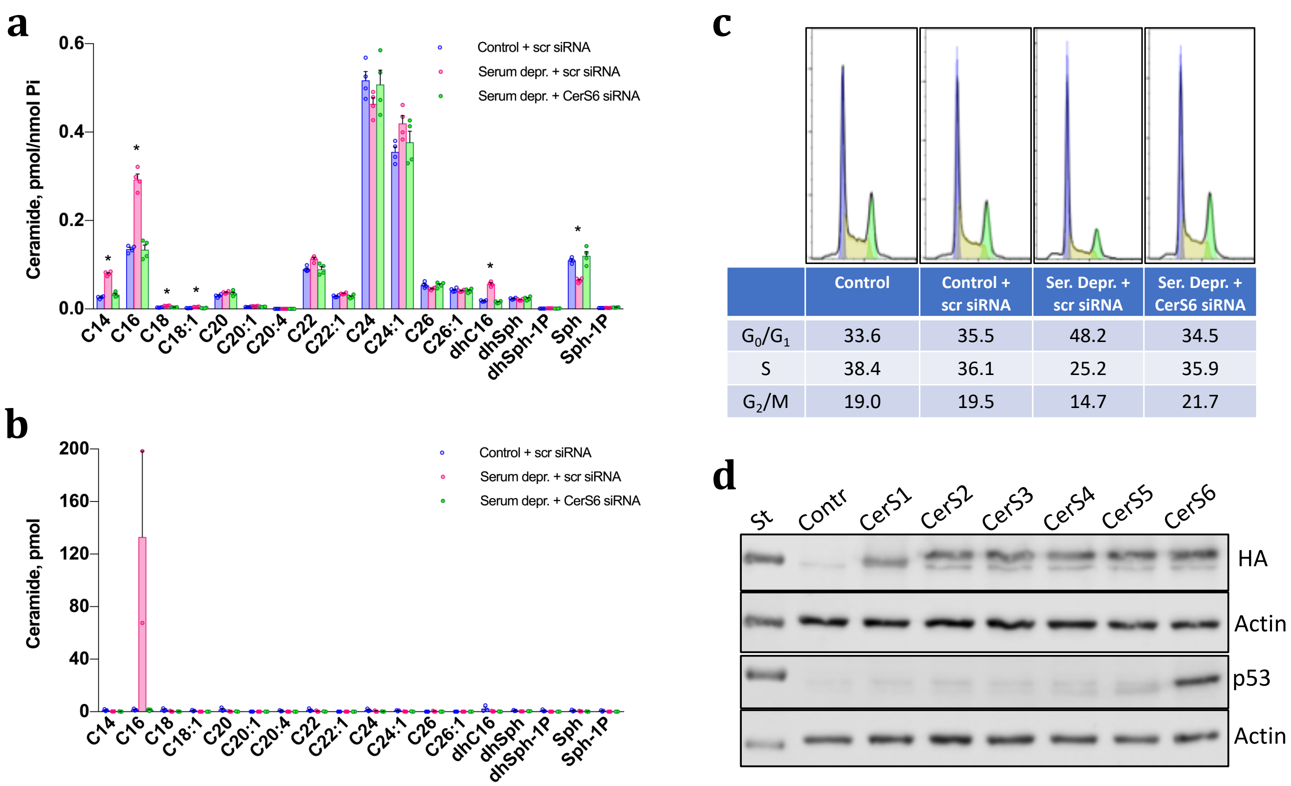

Supplementary Figure 10. Response to serum deprivation is mediated by CerS6 in HCT116 colorectal adenocarcinoma cells. (**a**) Serum deprivation results in elevation of C_{16}-ceramide in the HCT116 cells (48 hours after serum withdrawal); this elevation was not observed upon CerS6 silencing by siRNA (mean ±SE; n=4; *, P<0.0001, ANOVA). (**b**) p53 was pulled down in complex with C_{16}-ceramide from serum-starved cells but not from control or from serum-starved cells with siRNA silenced CerS6 (mean ±SE; n=3). (**c**) CerS6 siRNA silencing rescues G_0/G_1 cell cycle arrest upon serum deprivation. (**d**) Expression of CerS6, but not of CerS1-5 induced p53 elevation in HCT116 cells 48 h post-transfection.

a

b

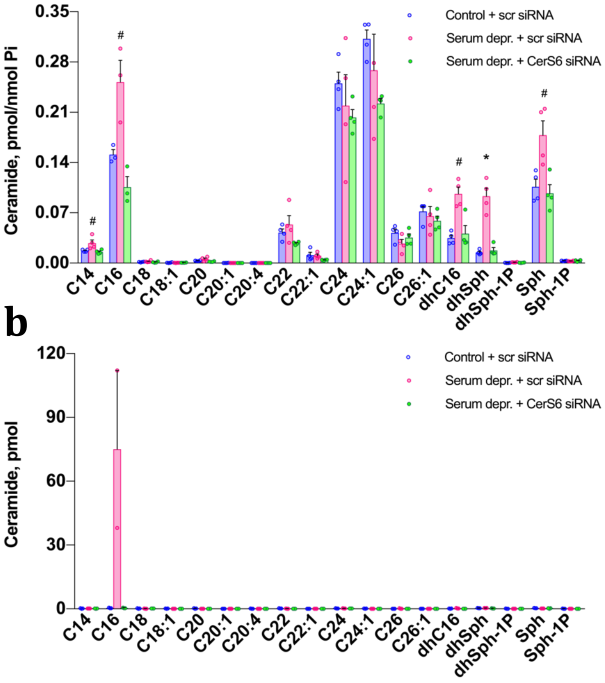

c

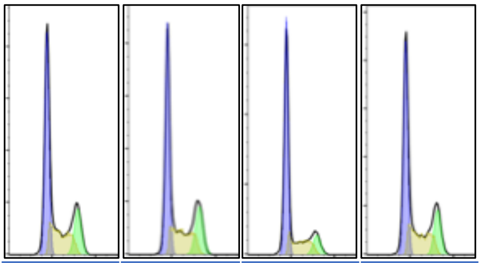

	Control	Control + scr siRNA	Ser. Depr. + scr siRNA	Ser. Depr. + CerS6 siRNA
G_0/G_1	52.9	52.1	63.8	51.4
S	28.4	28.7	23.0	29.6
G_2/M	19.4	17.9	10.9	17.3

Supplementary Figure 11. Response to serum deprivation in HepG2 cells is mediated by CerS6. (**a**) Serum deprivation elevates C_{16}-ceramide in the HepG2 cells (48 hours after serum withdrawal); this effect was abrogated upon CerS6 silencing by siRNA (mean ±SE; n=4; *, P<0.001; #, P<0.05, ANOVA). (**b**) p53 was pulled down in complex with C_{16}-ceramide from serum-starved HepG2 cells but not from control or serum-starved cells with siRNA silenced CerS6 (mean ±SE; n=3). (**c**) CerS6 siRNA silencing rescues G_0/G_1 cell cycle arrest upon serum starvation.

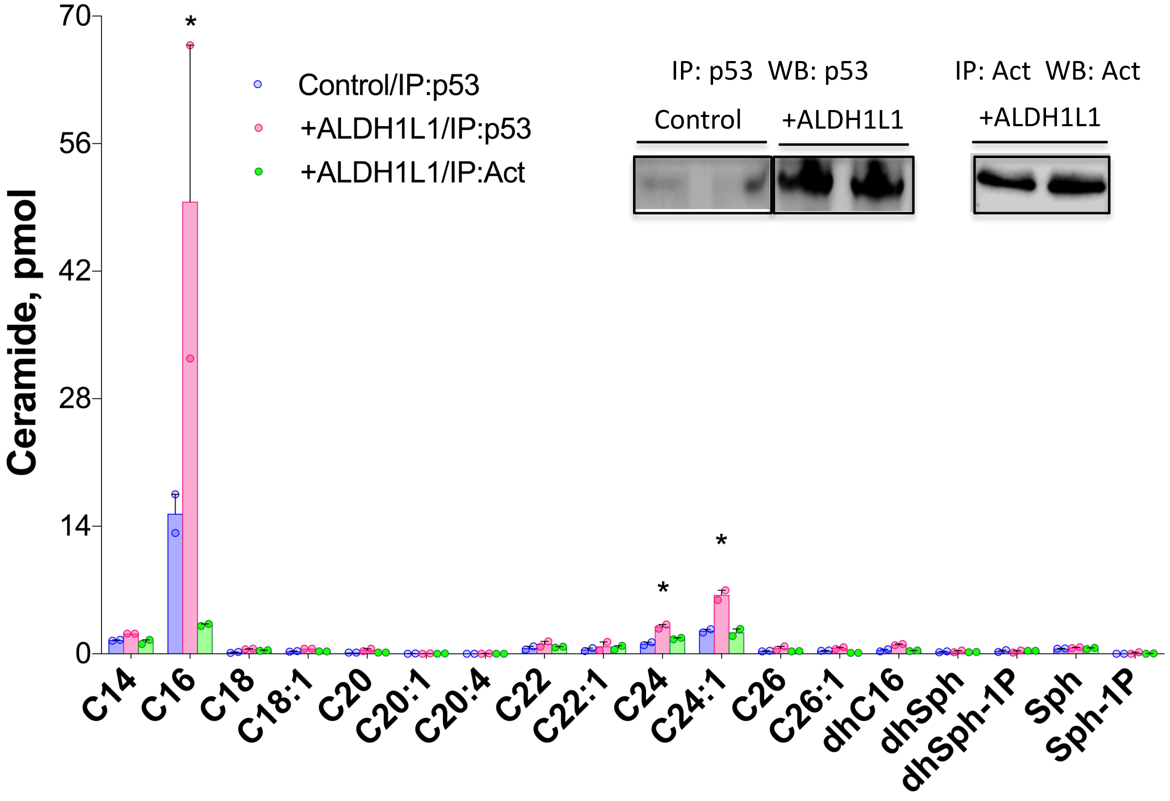

Supplementary Figure 12. p53 forms the complex with natural C_{16}-ceramide in the cells subjected to folate stress. Levels of ceramide species measured by LC/MS-MS in p53 and actin protein fractions pulled down from cell lysates using p53-specific or actin-specific antibody, correspondingly. Equal amounts of control A549 cells or cells transiently expressing a folate stress enzyme ALDH1L1 were analyzed. Lysates were divided into two portions which were used for p53 pull-down with p53-specific antibody and for actin pull-down with actin specific antibody. For each protein pull-downs were performed in duplicates. Data show the mean with the error bars representing SEM. Statistically significant differences (analyzed by ANOVA) are marked with asterisk (*, $P<0.05$). Inset shows the levels of corresponding proteins in pulled down fractions detected by immunoblotting.

Supplementary Table 1. Oligonucleotides used for site-directed mutagenesis.

p53 Substitution	Primers	Sequence
**S240K**	Sense	5′-CTACATGTGTAACAAGTCCTGCATG-3′
	Antisense	5′-CATGCAGGACTTGTTACACATGTAG-3′
**S240E**	Sense	5′-CTACATGTGTAACGAGTCCTGCATG-3′
	Antisense	5′-CATGCAGGACTCGTTACACATGTAG-3′
**S241A**	Sense	5′-CATGTGTAACAGTGCCTGCATGGG-3′
	Antisense	5′-CCCATGCAGGCACTGTTACACATG-3′
**S241K**	Sense	5′-CTACATGTGTAACAGTAAGTGCATGG-3′
	Antisense	5′-CCATGCACTTACTGTTACACATGTAG-3′
**S241E**	Sense	5′-CTACATGTGTAACAGTGAGTGCATGGG-3′
	Antisense	5′-CCCATGCACTCACTGTTACACATGTAG-3′

References

1. Levy, M. & Futerman, A.H. Mammalian ceramide synthases. *IUBMB Life* **62**, 347-56 (2010).
2. Mullen, T.D., Hannun, Y.A. & Obeid, L.M. Ceramide synthases at the centre of sphingolipid metabolism and biology. *Biochem J* **441**, 789-802 (2012).
3. Schiffmann, S. et al. Ceramide metabolism in mouse tissue. *Int J Biochem Cell Biol* **45**, 1886-94 (2013).
4. Petrache, I. et al. Ceramide synthases expression and role of ceramide synthase-2 in the lung: insight from human lung cells and mouse models. *PLoS One* **8**, e62968 (2013).
5. Ogretmen, B. Sphingolipid metabolism in cancer signalling and therapy. *Nat Rev Cancer* **18**, 33-50 (2018).

Figure 1b Full Scans, Top panel: A549 cells

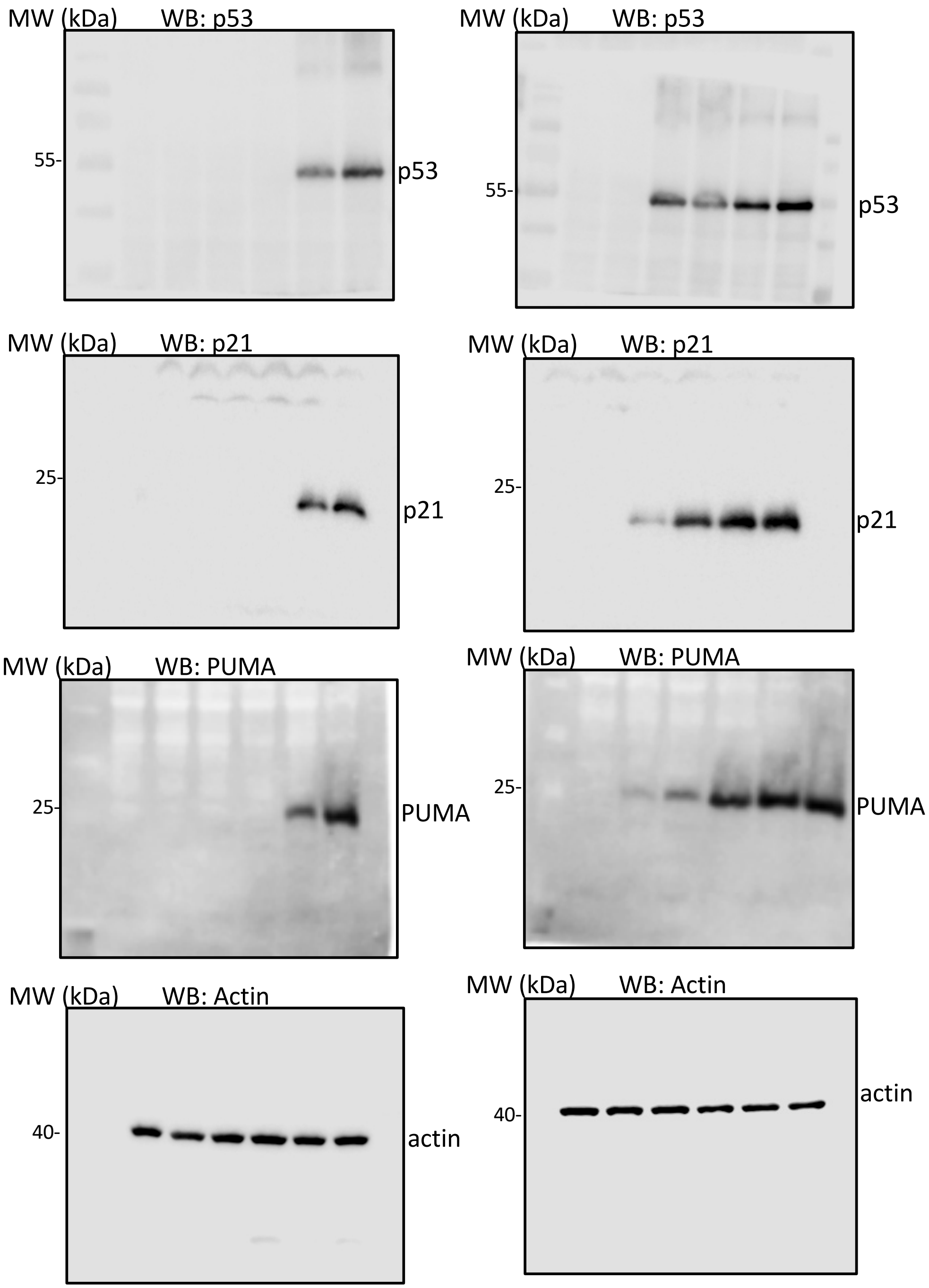

Figure 1b Full Scans, Middle panel: HepG2 cells

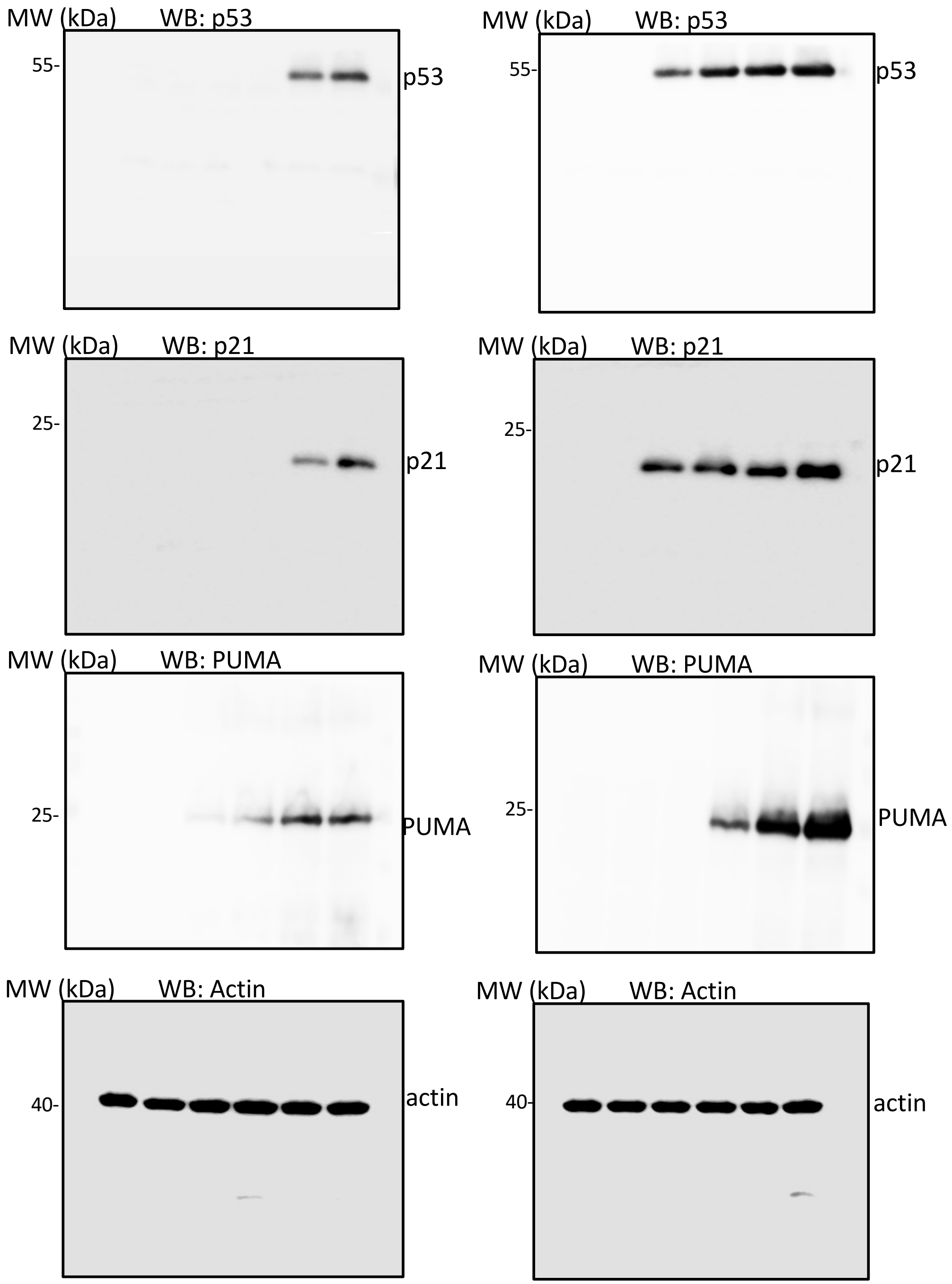

Figure 1b Full Scans, Bottom panel: HeLa cells

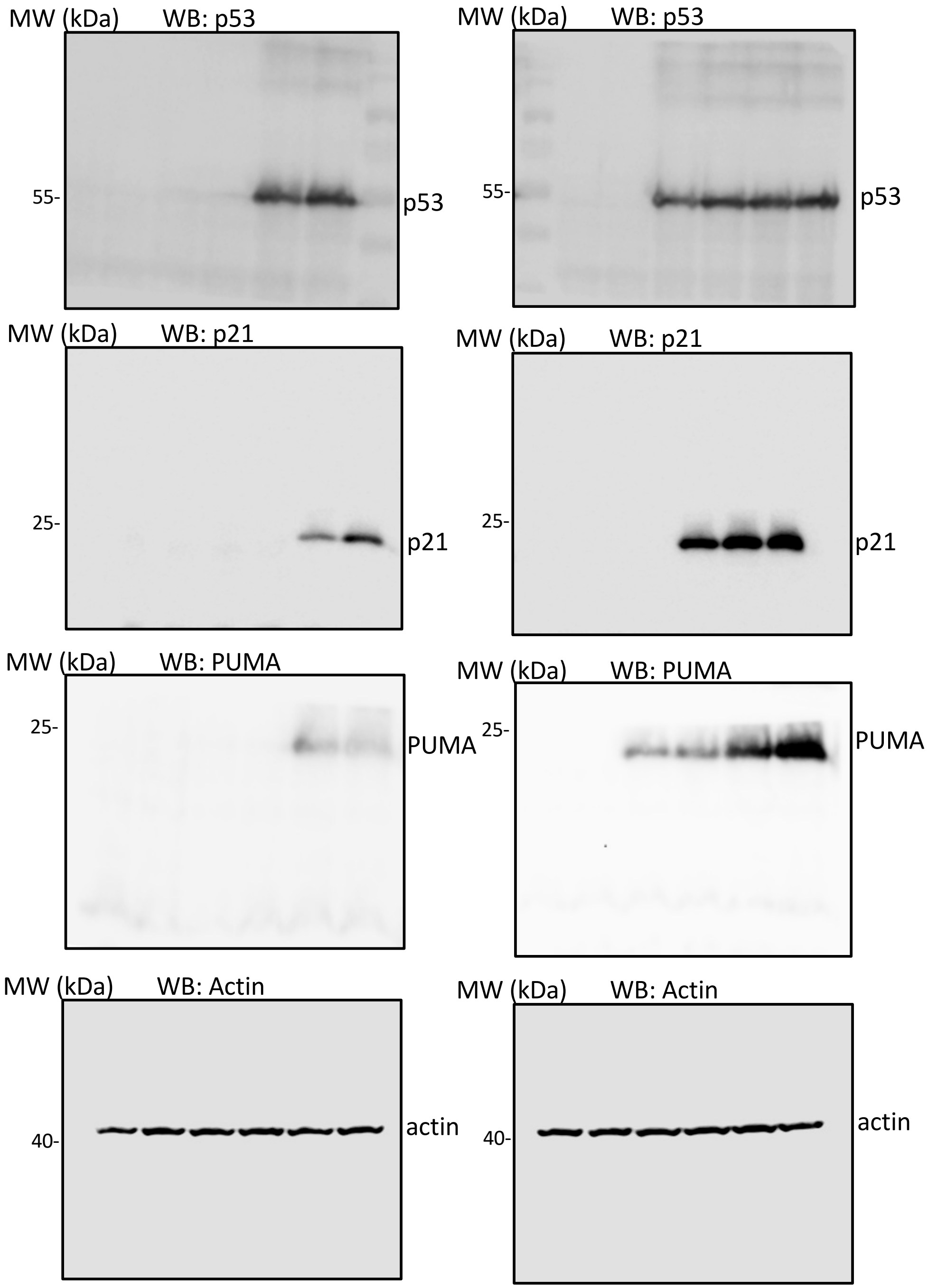

Figure 2a Full Scans

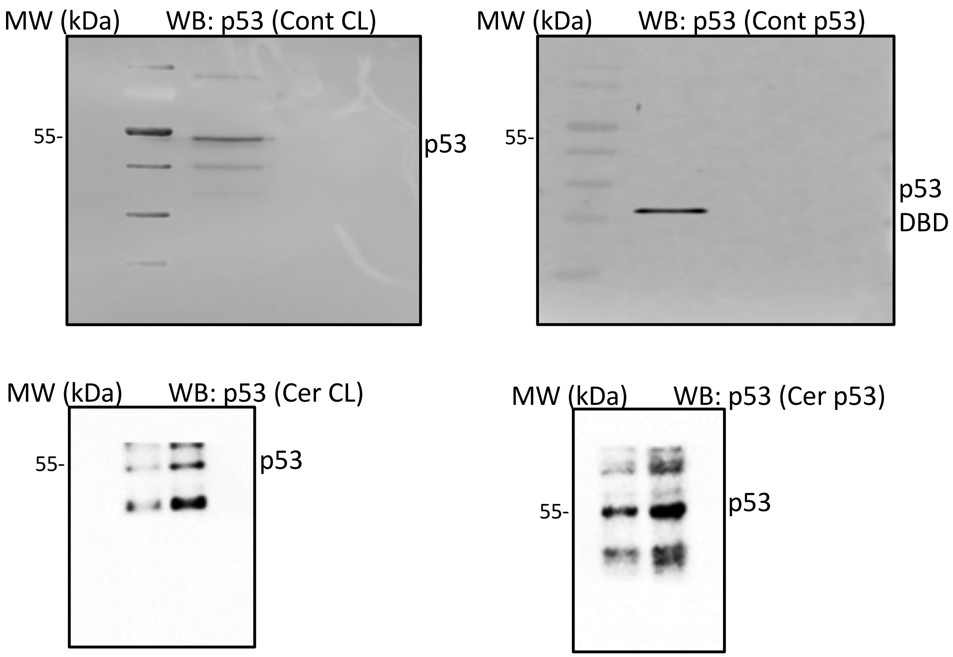

Figure 4d Full Scans, Right panel

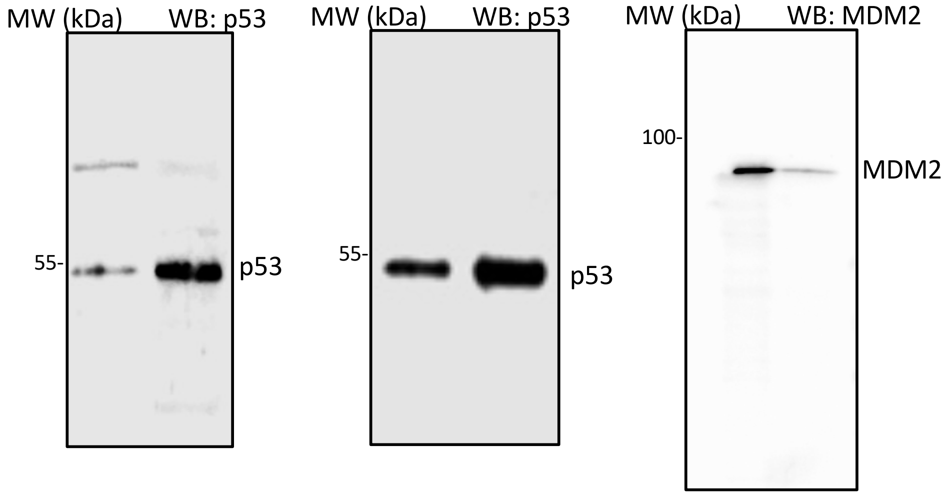

Figure 5a Full Scans

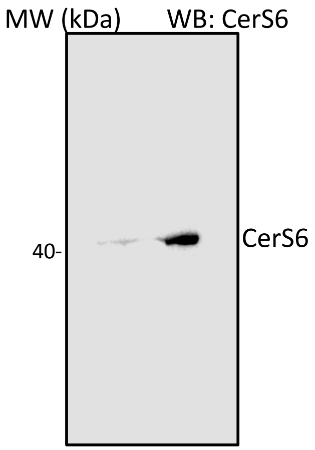

Figure 5b Full Scans

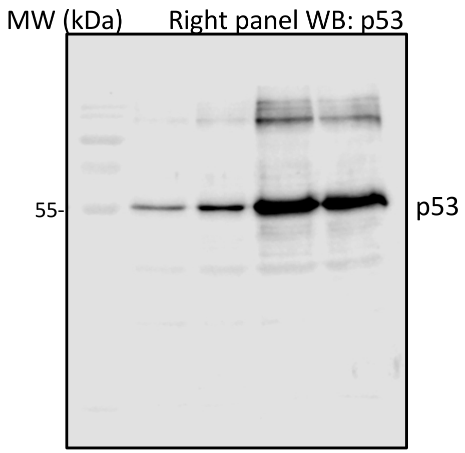

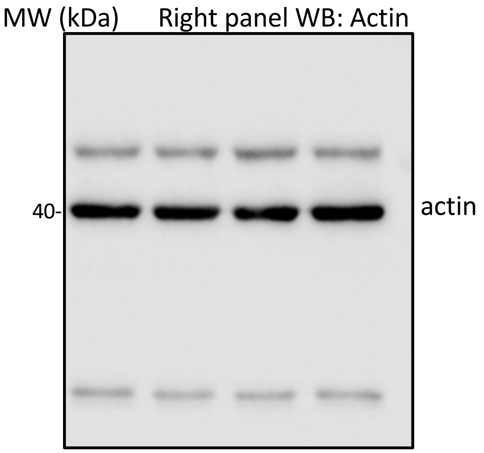

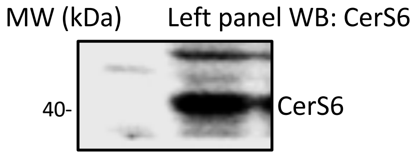

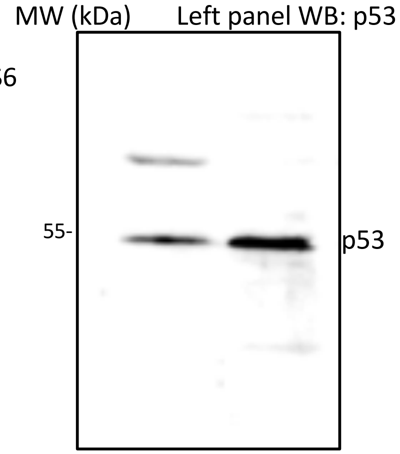

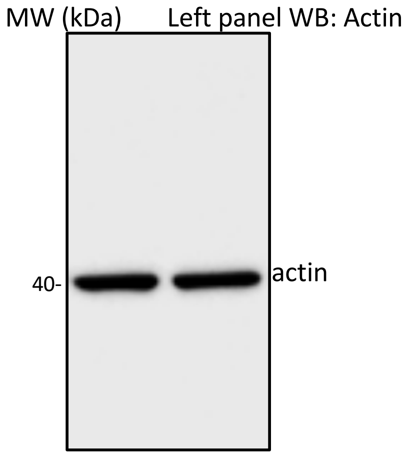

Figure 5e Full Scans

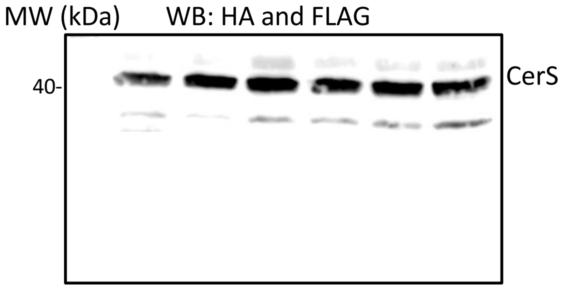

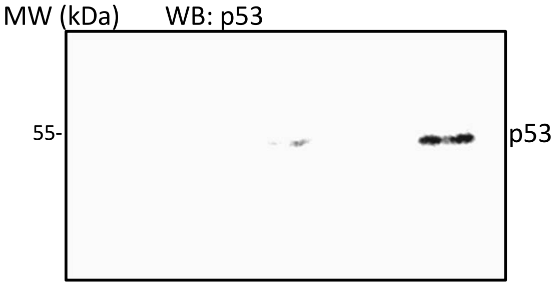

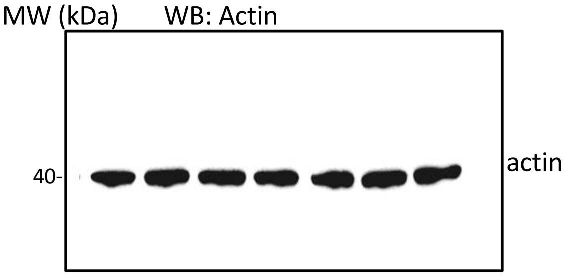

Figure 6a Full Scans, Top panel: HepG2 cells

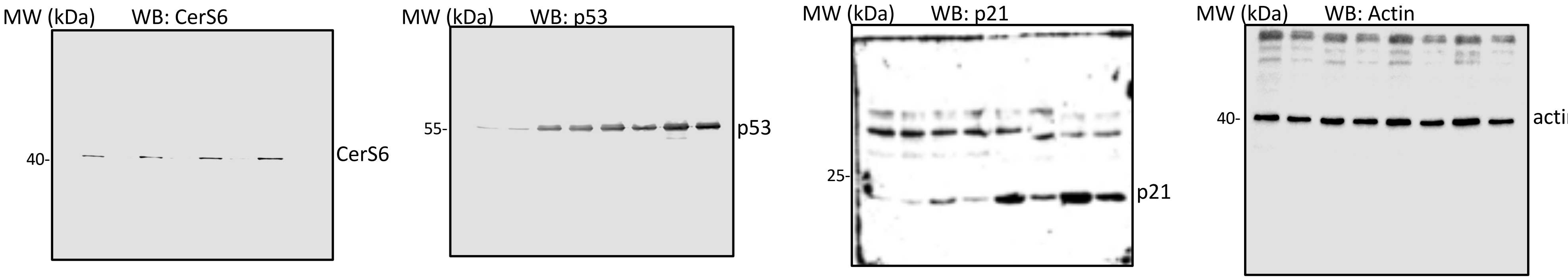

Middle panel: A549 cells

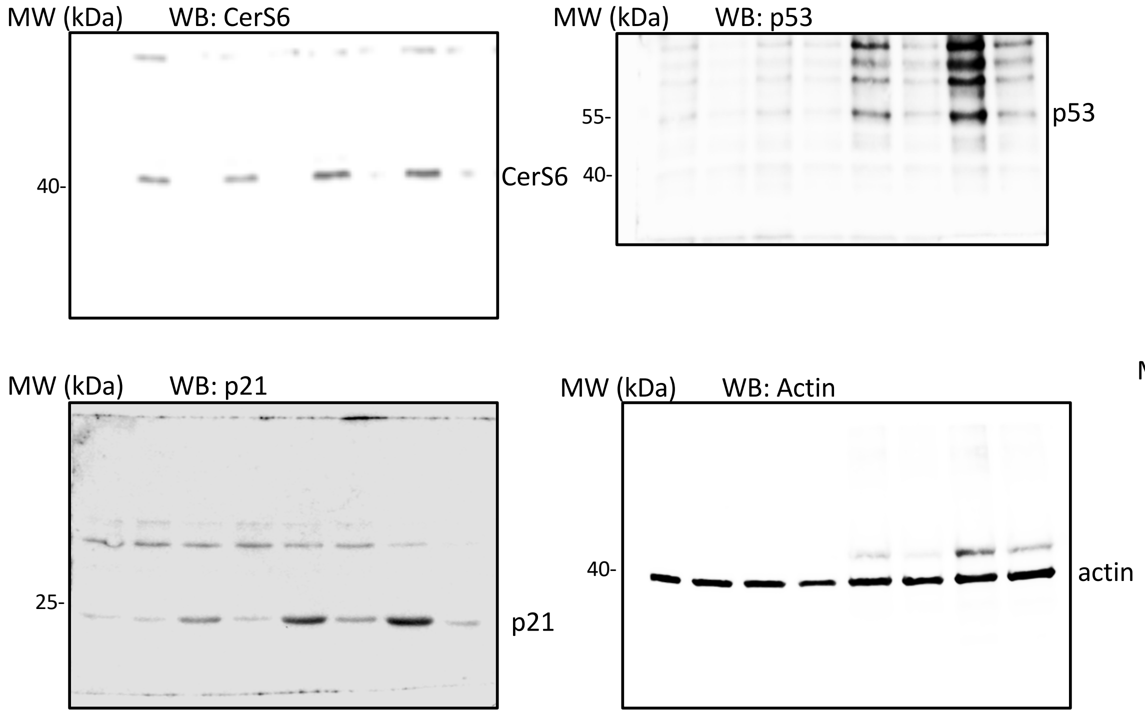

Bottom panel: HCT116 cells

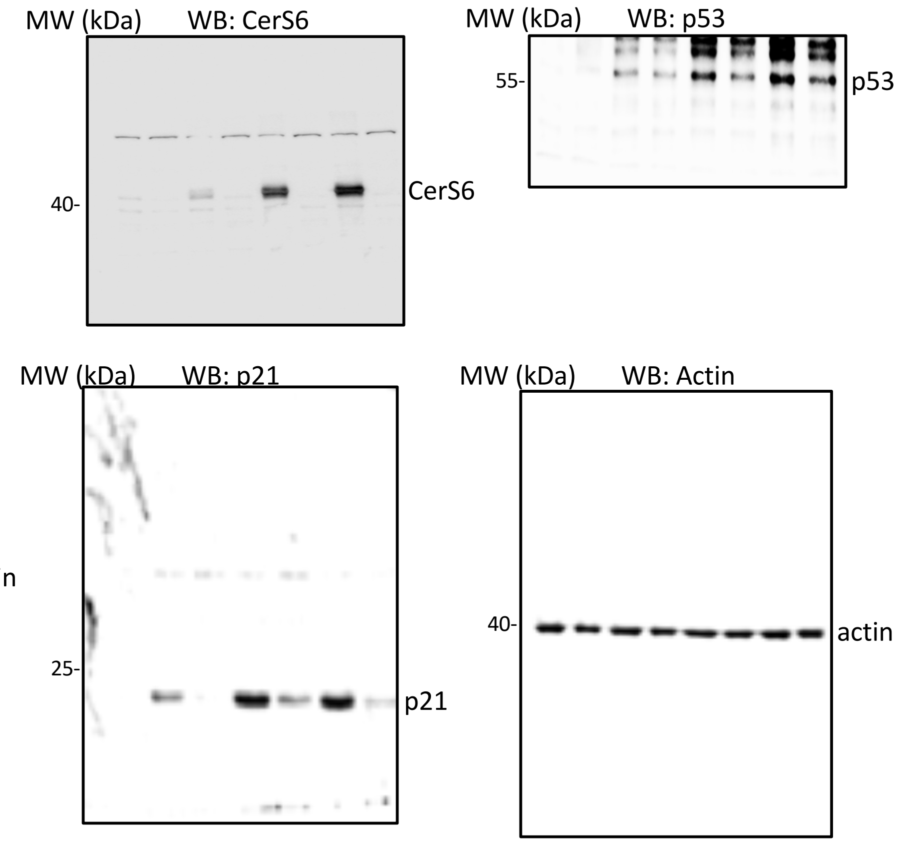

Supplementary Figure 4a Full Scans: Top panel

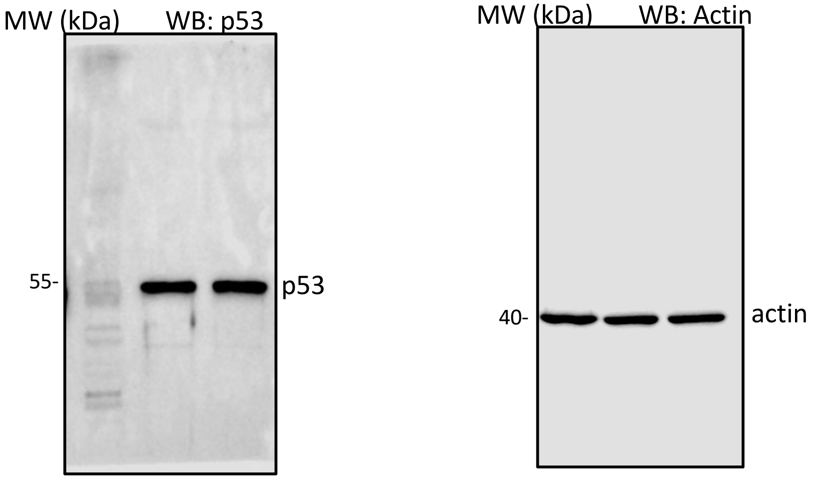

Supplementary Figure 5 Full Scans

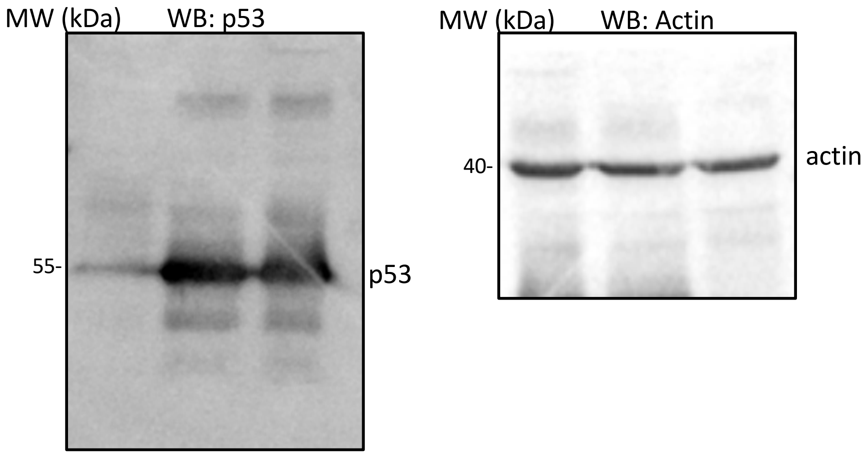

Supplementary Figure 4b Full Scans

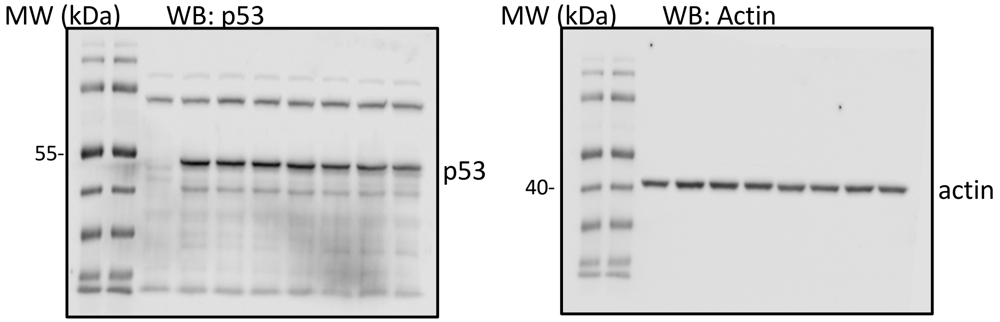

Supplementary Figure 8a Full Scans

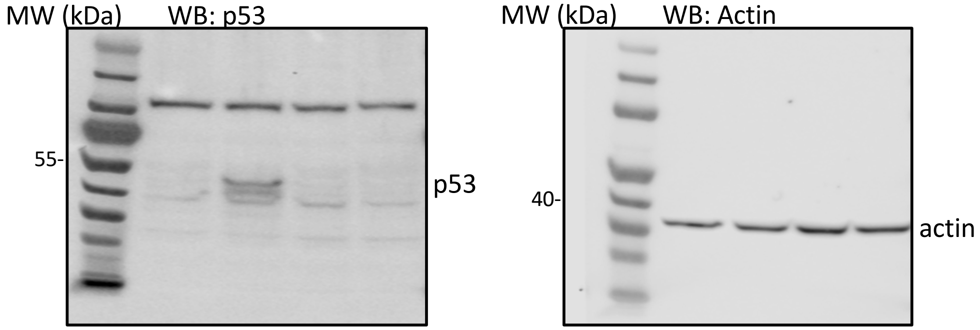

Supplementary Figure 8b Full Scans

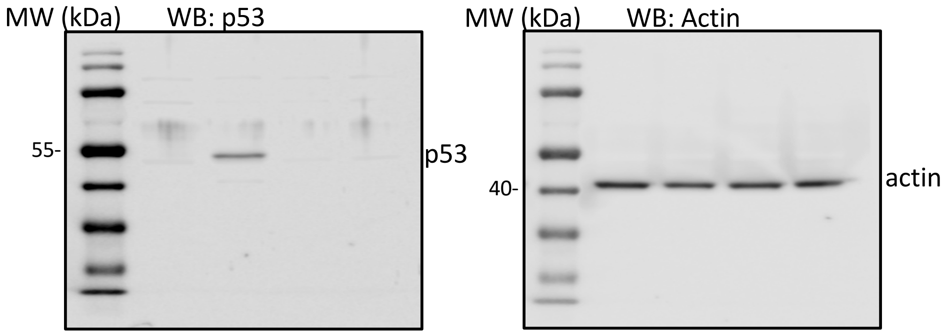

Supplementary Figure 9 Full Scans

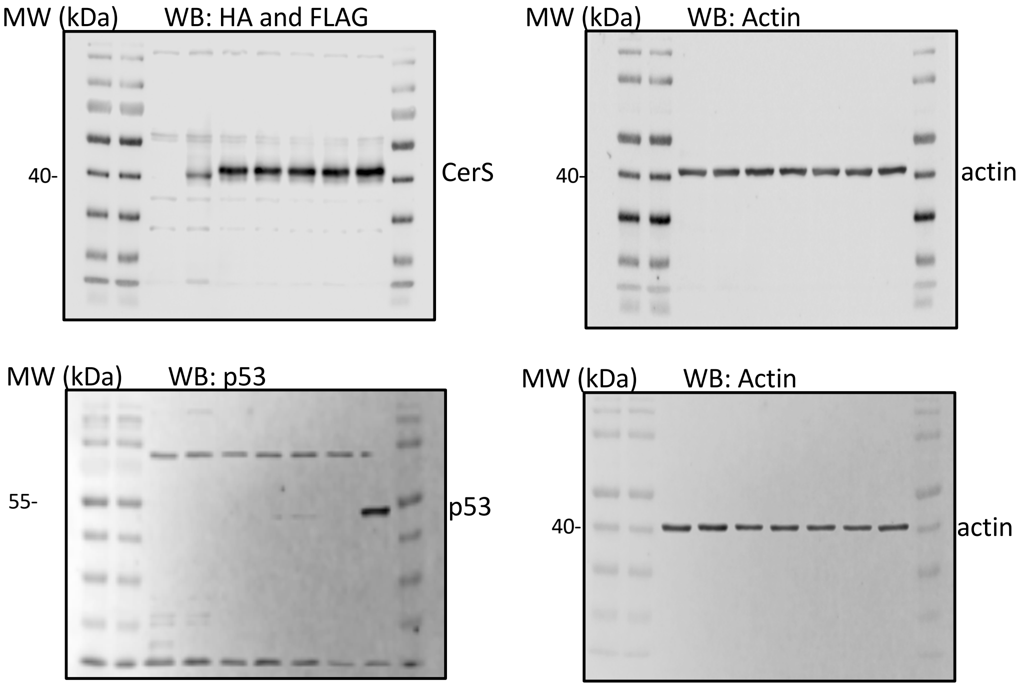

Supplementary Figure 10 Full Scans

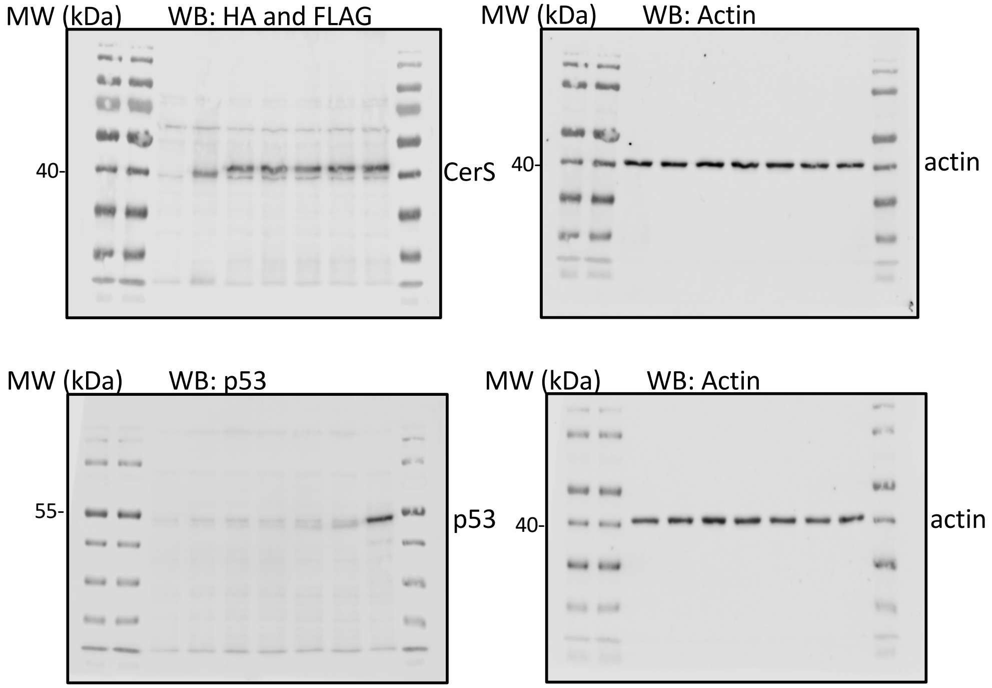

Supplementary Figure 12 Full Scans

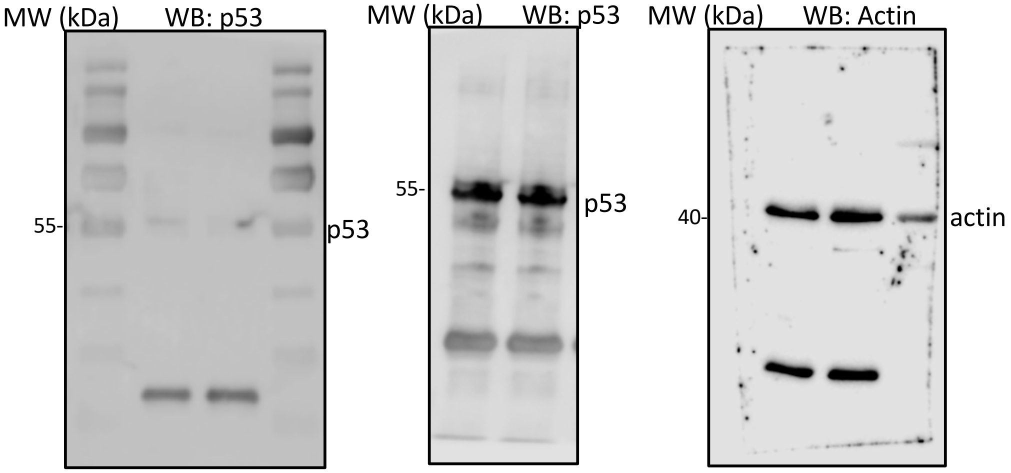

Supplement: Supplementary file 1 — Supplementary Information [file 41467_2018_6650_MOESM1_ESM.pdf]
